# Adding trans-abdominal elastography to the diagnostic tool for an ileal gastrointestinal stromal tumor: a case report

**DOI:** 10.1186/s12880-019-0385-6

**Published:** 2019-11-15

**Authors:** Wan-Ching Lien, Po-Chu Lee, Min-Tsan Lin, Chih-Heng Chang, Hsiu-Po Wang

**Affiliations:** 1 Department of Emergency Medicine, National Taiwan University Hospital and National Taiwan University, Taipei, Taiwan; 2 Department of Surgery, National Taiwan University Hospital and National Taiwan University, Taipei, Taiwan; 3 Department of Internal Medicine, National Taiwan University Hospital and National Taiwan University, No.7, Chung-Shan South Road, Taipei, 100 Taiwan

**Keywords:** Gastrointestinal stromal tumor (GIST), Elastography, Strain ratio, Strain histogram

## Abstract

**Background:**

Diagnosis of gastrointestinal stromal tumors (GISTs) in the distal small intestine is difficult by endoscopic ultrasound. This is the first reported case of an ileal GIST, which is diagnosed by transabdominal sonography and strain elastography.

**Case presentation:**

A 75 y/o woman presented with tarry stool and dizziness. No definite bleeder could be identified by esophagogastroduodenoscopy and colonoscopy. The transabdominal sonography revealed a large heterogeneous tumor involving the muscular layer of the ileum. Strain elastography showed the strain ratio was 6.51. Strain histogram was skewed to the blue side, and mean color value was 230.5, signifying a stiff tumor. GIST was highly suspected. The patient underwent laparoscope-assisted tumor excision and the histological examination confirmed a malignant GIST. The patient was discharged without postoperative event.

**Conclusion:**

Transabdominal strain elastography could play a role to discriminate small bowel GISTs and other submucosal tumors, especially in the location with difficulty in endoscopic ultrasound.

## Background

Gastrointestinal stromal tumors (GISTs), although rare, are the most common submucosal tumors (SMTs) of the gastrointestinal (GI) tract [1]. GISTs may arise from anywhere throughout the digestive tract, with 50–70% in the stomach and 30–45% in the small bowel (SB) [2, 3]. Tumor size, mitotic count, and tumor site of origin are the three key predictors of aggressive behavior and recurrence [4]. SB GISTs exhibit worse prognosis, especially in the ileum, compared to those arising in the stomach or colon [3, 5].

Clinical characteristics of GISTs vary according to different anatomic sites. SB GISTs may present with abdominal pain, a palpable mass, intestinal obstruction, or bleeding although some are insidious [6]. Endoscopic ultrasound (EUS) can be used to differentiate GISTs (hypoechoic solid masses) from other SMTs, such as lipomas (highly echoic masses), cysts (anechoic masses), and vessels [6], although the mass would be sometimes misdiagnosed as ectopic pancreas, neuroendocrine tumor or solid pseudopapillary tumor [7, 8]. EUS-guided fine-needle aspiration (EUS-FNA) is a key component in the diagnosis of GIST [3, 7]. However, obtaining adequate specimens is difficult in some cases because of tumor size and location [7].

Elastography has been widely used for diagnosis of breast cancer, thyroid nodules and liver fibrosis [8]. There are two frequently used techniques: strain elastography (SE) and shear-wave elastography (SWE). SWE uses an impulse created by a focused ultrasound beam, which measures the propagation speed of shear waves within the tissue and presents as average speed or kilopascals in a limited area [8, 9]. In contrast, stress is applied by repeated manual compression of the transducer, shape change of the target tissue relative to the surrounding tissue is measured and displayed in color in SE [8, 9]. Red color indicates a soft tissue and blue indicates a hard tissue. Strain ratio is calculated as: average strain in reference tissue/average strain ROI in target tissue. In strain histogram analysis, tumor stiffness is calculated as mean pixel color values of a single region of interest (ROI) within the tumor, ranging from 0 to 255 (from red over green to blue). The higher the strain ratio and the mean histogram value, the stiffer the tumor. Notably, strain histograms are independent of reference ROIs, even if the surrounding tissue is inhomogeneous [10].

Evidence regarding the applications of elastography for GISTs are mainly with endoscopic devices. Tsuji et al reported that EUS-elastography could be used to differentiate gastric GISTs with other SMTs because of its stiffness [7]. Few data exists regarding transabdominal elastography for SB GISTs [11].

Our case is the first reported ileal GIST, which was diagnosed with transabdominal ultrasonography and SE preoperatively. The characteristics of elastography are reported with a review of the literature. This study was approved by the Institutional Review Board of the National Taiwan University Hospital and an informed consent was obtained from the patient.

## Case presentation

A 75 y/o woman visited the emergency department because of tarry stool and dizziness for one day. Progressively exertional dyspnea was noted 2 weeks before the visit. Her medical history included coronary artery disease.

At arrival, her physical examinations revealed pale conjunctivae, and soft abdomen without tenderness. Laboratory data showed normocytic anemia with hemoglobin 7.6 g/dL, and positive stool occult blood. Others were unremarkable. Esophagogastroduodenoscopy and colonoscopy were done, but no definite bleeder could be identified. Small bowel bleeding was suspected.

**Fig. 1:**
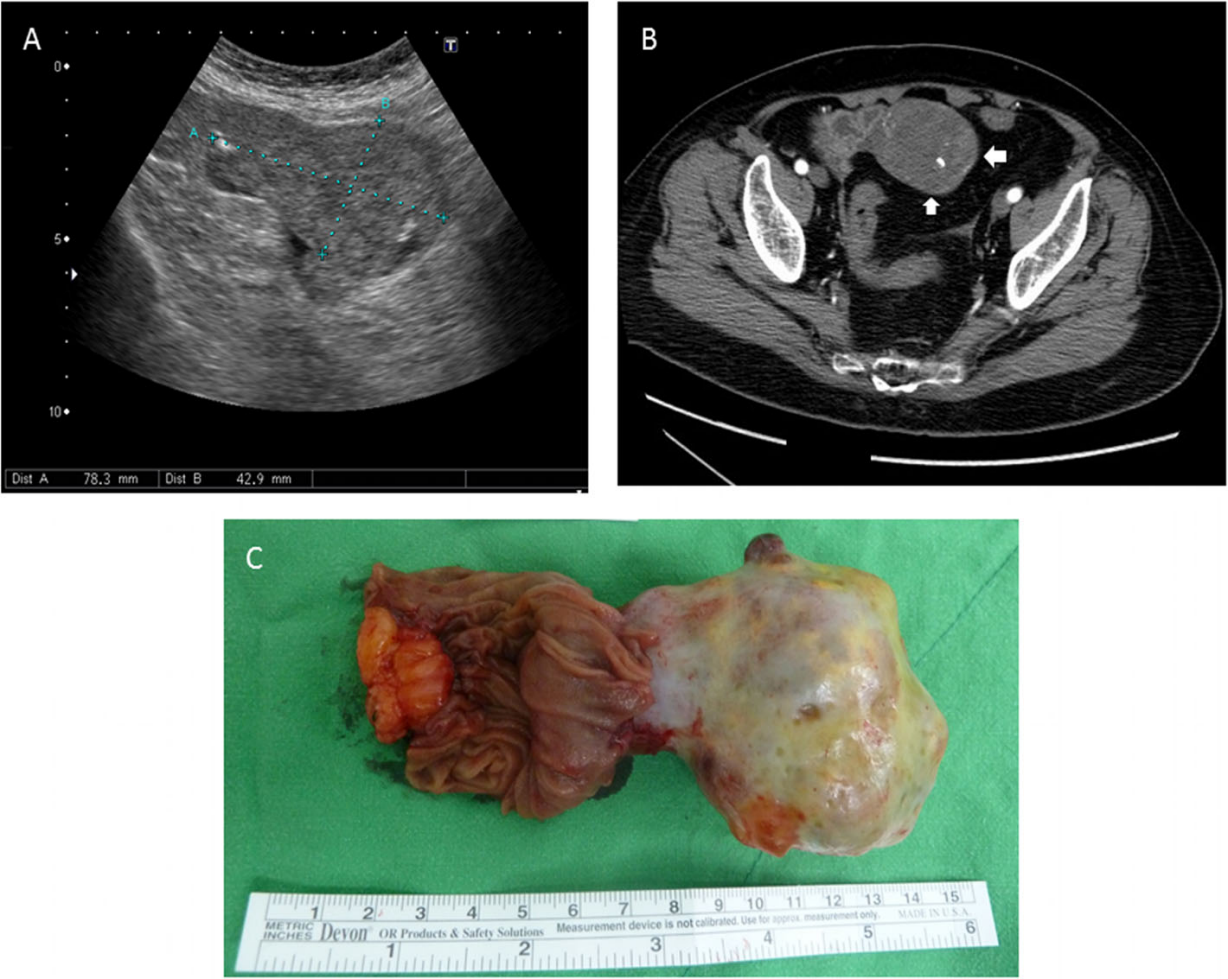
An ileal tumor. **a**, Transabdominal ultrasonography shows a 7.8 cm × 4.2 cm heterogeneous lesion over ileum. **b**, A large lobulated tumor with heterogeneous enhancement at the proximal ileum. **c**, A well-defined fleshy tumor at the proximal ileum was resected

**Fig. 2:**
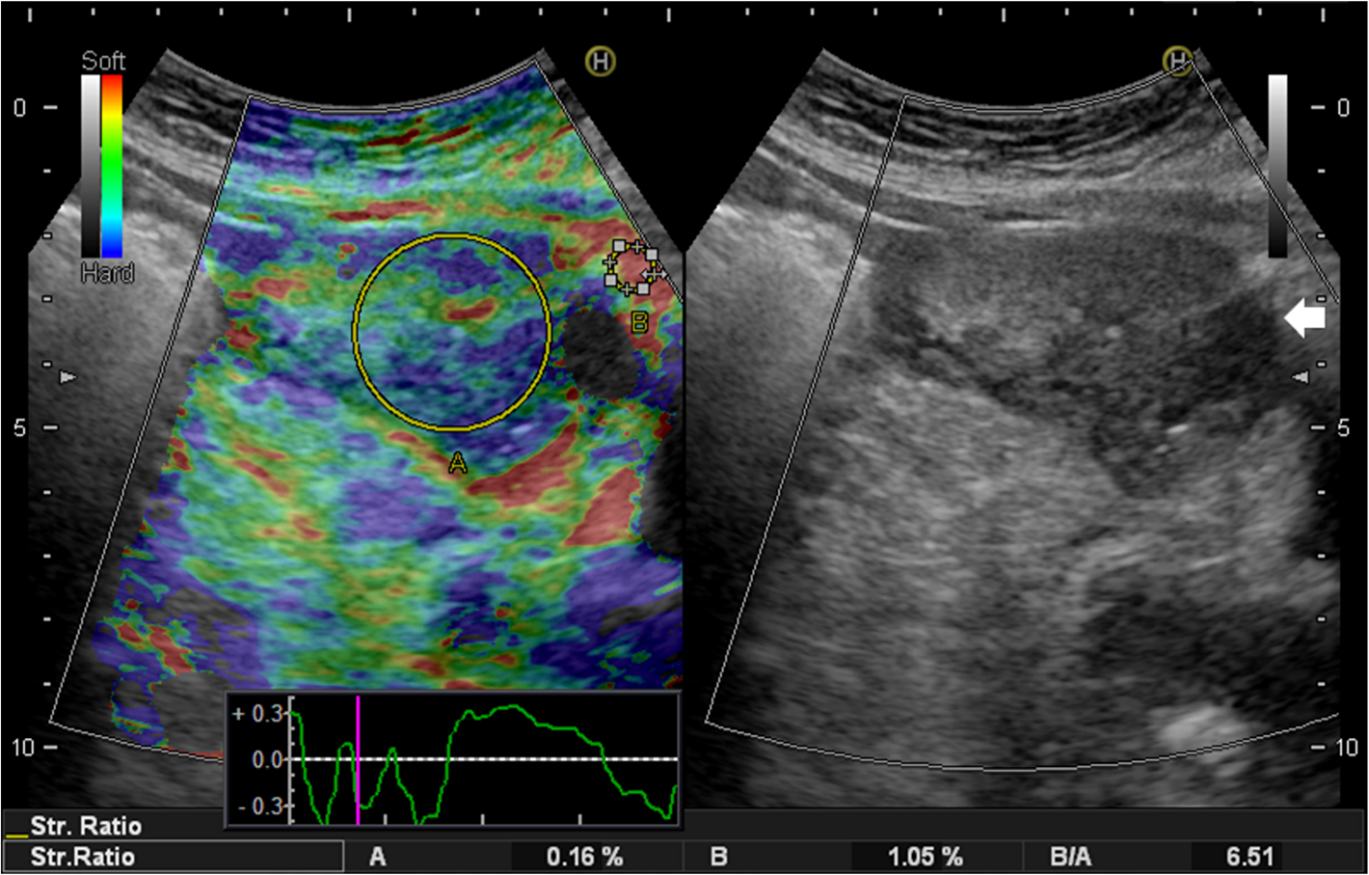
Image showing a split-screen image with strain elastography on the left side and B-mode on the right side. The B-mode image shows a solid, circumscribed, hypoechoic tumor with some necrosis (arrow). Red color indicates a soft tissue and blue indicates a hard tissue in strain elastography. The tumor with a mosaic color pattern of mainly green and blue, indicating a relatively hard tumor. Region of interest (ROI) A is placed within the tumor and ROI B is placed in adjacent fatty tissue. The strain ratio of the tumor was 6.51, indicating a hard tumor

**Fig. 3:**
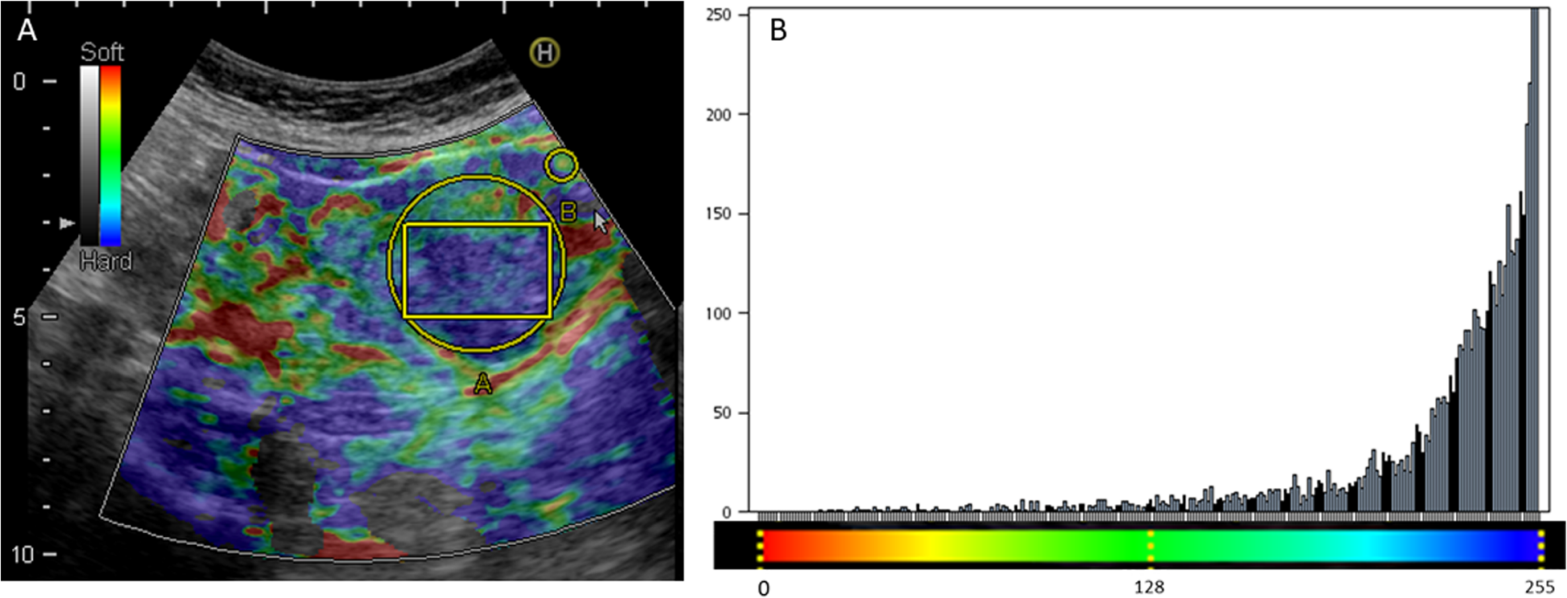
Strain histogram analysis of the ileal tumor. **a**, The strain elastogram shows a predominantly blue tumor. Strain histogram analysis was performed from a single ROI within the tumor (ROI A). **b**, The strain histogram is displayed as a bar chart, with the pixel color values on the x-axis, and the number of pixels of a certain color pr. 1000 pixels within the ROI on the y-axis. The mean color value of the tumor is 230.5, signifying a stiff tumor

Transabdominal sonography revealed a 7.8 cm × 4.2 cm heterogeneous solid tumor involving the muscular layer of ileum, suspected a GIST (Fig. 1a). Elastography was applied, using a Hitachi Noblus system (Hitachi, Tokyo, Japan) with a C251-probe with a bandwidth of 1–5 MHz. It showed a mosaic color pattern of mainly green and blue in correspondence with the thickening of the bowel wall. The strain ratio was 6.51, comparing with the reference ROI (Fig. 2). The color distribution of the strain histogram was skewed to the blue side, and the mean color value was 230.5, signifying a stiff tumor (Fig. 3). Abdominal computed tomography (CT) showed a lobulated tumor with heterogeneous enhancement, consistent with a GIST (Fig. 1b).

**Fig. 4:**
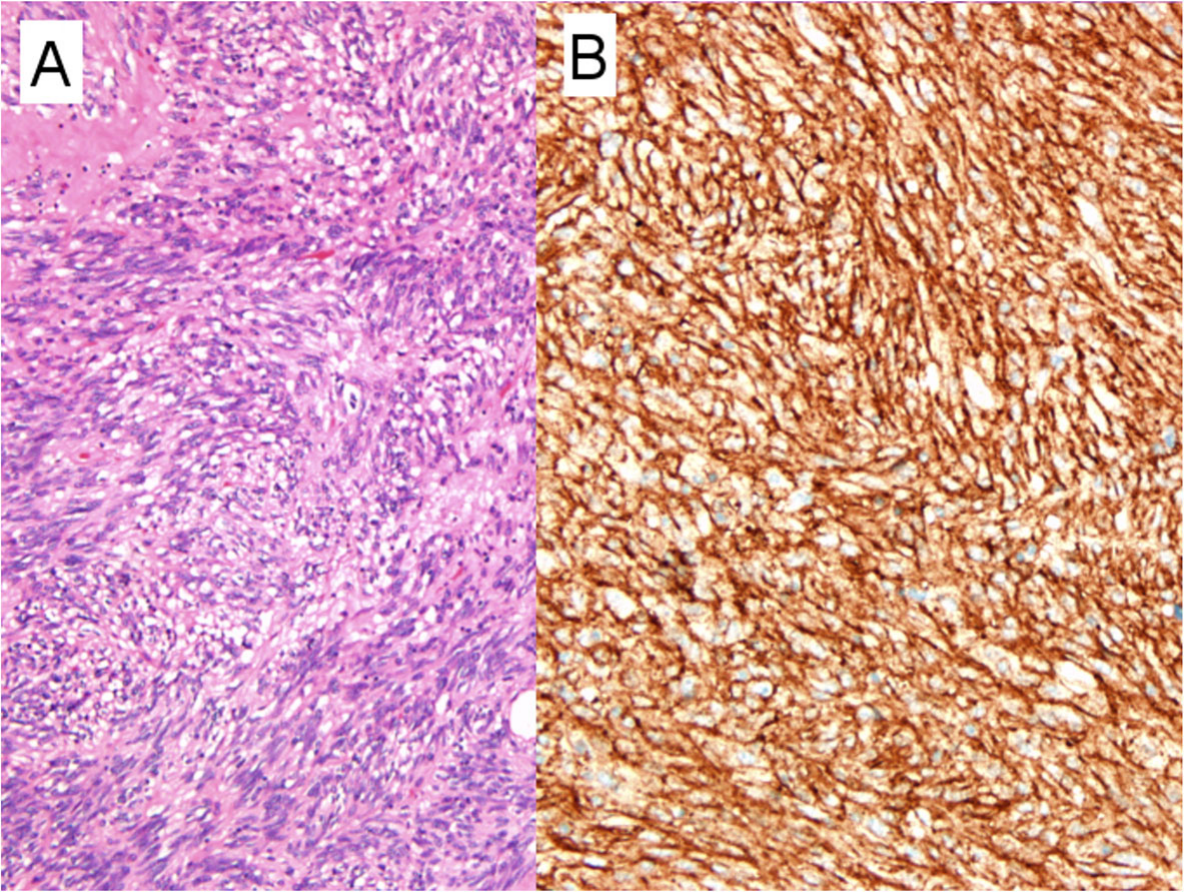
The histologic examination. **a**, Hematoxylin–eosin, × 100 magnification. Aggregates of neoplastic cells with high cellularity, marked pleomorphism and mitosis. **b**, Immunohistochemical staining of CD117 (c-kit), × 200 magnification. Positive staining for CD117

The patient underwent laparoscope-assisted tumor excision. The tumor presented as a pedunculated mass extending from the ileal wall (Fig. 1c). Grossly, it was fleshy with hemorrhagic necrosis. The histological examination revealed a malignant GIST, spindle cell type (Fig. 4a), with high cellularity, positive c-kit immunohistochemical stain (Fig. 4b) and a high mitotic rate (> 5/50 high power fields), corresponding to WHO classification 6a. She was discharged uneventfully. Imatinib was prescribed at the follow-up out-patient clinic.

## Discussion and conclusions

EUS-FNA has been demonstrated to have high diagnostic yield for GISTs at upper GI tract [12]. However, GISTs at the distal part of the SB may be not amenable. In addition, the number of specimen obtained in such invasive procedures may be inadequate. Bleeding or perforation may occur after procedures [7]. In conventional imaging modalities, GISTs may exhibit similar characteristics as other SMTs that pre-operative discrimination may be difficult.

Elastography is a sonographic technique that depicts elasticity of tissues [13]. SE and SWE are frequently used techniques, with differences in their forces and imaging methods [9]. Some authors reported that the image acquisition and interpretation of SE could be influenced by the operater’s experience. An increase in artifacts in SWE could be caused by reflection and refraction of shear-wave [9]. However, the diagnostic accuracy was reported to be similar between SE and SWE [9], although some discrepancies exist because of target diameter and depth [14].

Recently, SE or SWE has been suggested as an adjunct tool for assessing GI diseases [15–17]. However, the data is still limited at transabdominal elastography [11, 18]. Previous study reported that gastric GIST was a harder tumor than other SMTs in EUS elastography [7]. There is the first reported case of a SB GIST at transabdominal ultrasonography, in conjunction with SE and strain histogram. Transabdominal sonography demonstrated the location of the tumor, and the characteristics of stiffness was detected by transabdominal SE. After tumor excision, malignant GIST was also confirmed at the histologic examination. It suggested that transabdominal SE may be helpful for preoperative assessment for SB tumors. In addition, the characteristics of the GIST in transabdominal SE was similar to that reported in EUS SE [7].

Inhomogeneous surrounding tissue, and reference ROIs located in different depths are the challenges for strain ratio measurements [19], especially in transabdominal elastography for GI tract. Strain histogram analysis is independent of reference ROIs, that tumor stiffness can be calculated as mean pixel color values within the tumor [10]. A current study suggested strain histogram analyses exhibit the comparable diagnostic accuracy with strain ratio analyses [10]. In this case, we demonstrate the characteristics of the GIST in strain ratio and histogram analyses. There is an agreement between these two analyses, suggestive of a hard tumor. It was confirmed by the histologic examinations with high cellularity. In the present case, SE could provide more information regarding the characteristics of the SB tumor.

A focal lesion with increased stiffness at elastography is usually suggestive of malignancy in breast, and thyroid diseases [8, 19]. Strain ratio more than 2.5 indicates of malignant breast lesions [20]. Whether it could be applied into GISTs is still unclear that further investigations are needed.

In conclusion, our case well demonstrates the use of transabdominal SE for an ileal GIST. Transabdominal SE could play a role to discriminate GISTs from other SMTs, especially in the location that performing EUS-FNA is difficult.

## Data Availability

The datasets used during the current study are available from the corresponding author on reasonable request.

## Abbreviations

CT: Computed tomography
EUS: Endoscopic ultrasound
EUS-FNA: Endoscopic ultrasound fine-needle aspiration
GI: Gastrointestinal
GISTs: Gastrointestinal stromal tumors
ROI: Reference region of interest
SB: Small bowel
SE: Strain elastography
SMTs: Submucosal tumors
SWE: Shear-wave elastography
